# Valorization and characterization of corn by-product polyphenols through green extraction technologies

**DOI:** 10.3389/fnut.2023.1107067

**Published:** 2023-05-09

**Authors:** Neelam Faiza, Ali Imran, Muhammad Umair Arshad, Muhammad Sajid Arshad, Mohd Asif Shah

**Affiliations:** ^1^Department of Food Sciences, Government College University, Faisalabad, Pakistan; ^2^Department of Economics, Kebri Dehar University, Kebri Dehar, Ethiopia; ^3^Division of Research and Development, Lovely Professional University, Phagwara, Punjab, India

**Keywords:** agro-industrial waste, corn silk, mature, polyphenols, green extraction technologies

## Abstract

The amount of food waste throughout the world has become quite alarming and is contributing to lower food resources. The study aimed to extract and characterize the polyphenols from corn silks at immature and mature stages through conventional and green extraction techniques. Purposely, corn silks, which are some of the by-products of corn, (*Zea mays* L.) were collected and subjected to proximate analysis including moisture, ash, protein, fiber, and minerals. Secondly, the antioxidants from both immature and mature corn silks were extracted by techniques involving supercritical and ultrasound extraction alongside conventional extraction. The results displayed a promising quantity of protein and fiber along with calcium, magnesium, sodium potassium, and copper. Among the extraction techniques, supercritical extraction at 3,000 Pa acquired the highest total phenolic contents (TPC), total flavonoids (TF), 2, 2-diphenylpicrylhydrazyl (DPPH), ferric-reducing antioxidant power (FRAP) activities as 128.08 ± 3.74 mg GAE/100 g, 86.73 ± 2.75 mg CE/100 g, 106.73 ± 5.10%, and 73.52 ± 2.33 μM Fe + 2/g, respectively, followed by the ultrasound and conventional extraction techniques. Between the immature and mature corn silks, the highest antioxidant activity was displayed by immature corn silks.

## Introduction

Agro-industrial waste poses huge environmental and financial hurdles at the point of disposal. However, they hold rich phytochemistry with special reference to polyphenols that may prove effectual against various lifestyle-related ailments. In this context, waste valorization is among the leading strategies to ensure food security by minimizing loss and recycling beneficial components that may be further utilized as therapeutic agents (1). Among the various waste-generating crops, corn (*Zea mays* L.) has vital importance primarily due to its higher consumption and rich phytochemistry. Parts of the corn (*Zea mays* L.) plant hold promising nutritional and therapeutic potential owing to its strong polyphenol contents (2, 3). Amongst the different waste products of corn, the bundles of silky, hair-like, long, and yellow in color strands, which can be found on the top of the immature or mature corn, are called corn silks and they hold significant importance. Corn silk is usually treated as waste; however, recent investigations unveiled its nutritional and functional profile with special reference to fiber, minerals, and antioxidants (4). Research has also found that the stage of maturity also plays a pivotal role in its antioxidant profile (5). Conventionally, these hair-like strands are utilized as a diuretic agent and are vital for providing ease for the passage of kidney stones or urinary bladder stones (6). Furthermore, Chinese people have been utilizing these corn silks for medical purposes and treating several ailments usually related to the kidneys (7). The therapeutic potential of corn silk has been attributed to its high polyphenolic contents with special reference to phenolic acids like ferulic acid, caffeic acid, and gallic acid. Moreover, the demand for natural antioxidants has been on the increasing side because of negative perceptions associated with synthetic antioxidants that are available in the market (8). All around the world, plants and their waste products are a potent source of natural antioxidants and help in combating many ailments (9–11).

Currently, green extraction technologies have been adapted widely for the extraction of high-value-added compounds from agro-industrial wastes (12). The utilization of novel technologies for polyphenol extraction can improve the extraction yield and therapeutic potential. Moreover, they are rated as affordable, safe, effective, and ecologically friendly alternatives, enabling the clean label status (13). Polyphenol extraction yield depends on the technique applied and novel green extraction technologies like ultrasound-assisted extraction, supercritical fluid extraction, microwave-assisted extraction, and pulse electric field extraction are considered the most efficient owing to their higher yield, extraction time, costs, and environmental impact. However, the success of the applied technique depends on several factors including the nature of raw material, chemical constituents, and processing parameters (14). The green technologies utilized for extraction help in efficient structural elucidation along with less solvent and time required for the completion of the procedures. Supercritical fluid extraction (SFE) uses CO_2_ to separate the components while ultrasonication uses the frequencies from sound waves to extract the required components. Considering the abovementioned facts, it is envisaged that there would be conversations regarding the impact of the different extraction techniques on the polyphenol extraction of corn silk and their structural elucidation. This study aimed to elucidate the nutritional composition of corn silk and its antioxidant profiling through different green extraction techniques like supercritical fluid extraction and ultrasonication alongside side conventional extraction for comparison.

## Materials and methods

### Procurement of raw material

For the current research, the corn was procured from the Ayub Agriculture Research Institute (AARI), Faisalabad, with a plant voucher number (GCBFA-3347), while the chemicals utilized for the HPLC standards and analytical procedures were procured from Merck (Germany) and Sigma-Aldrich (Japan).

### Raw material handling

The corn silks were removed from the corn fruits and washed thoroughly with distilled water to remove any impurities. After washing the corn silks, they were dried in a hot air oven at 50 to 60°C for approximately 48 h. Finally, the oven-dried sample of corn silks was ground into a powder form with the help of a small laboratory grinder (Panasonic, Japan, Model MJ-W176P) and stored at an optimum temperature for further use in the research.

### Proximate analysis

The proximate analysis of the corn silks was carried out by the determination of the moisture content, protein, lipid (fat), ash, and dietary fiber by following the protocol of AACC (15). However, the nitrogen free extract (NFE) was calculated at the end through the subtraction method.

### Mineral analysis

The corn silk powder sample was then evaluated for its mineral content consisting of minerals like Na (sodium), K (potassium), Ca (calcium), Zn (zinc), Fe (iron), and Mg (magnesium) following the guidelines of AOAC (16). The first two minerals were determined by Flame Photometer-410 (Sherwood Scientific Ltd., Cambridge) while the remaining were estimated by Atomic Absorption Spectrophotometer (Varian AA240, Australia).

## Preparation of extracts

### Experimental design

We utilized the following conditions for the different extraction techniques owing to the experimental techniques that were already approved appropriately in a preliminary trial. Moreover, the extraction of polyphenols from waste materials had already been carried out in our laboratories and the following conditions had been proven suitable so we used them in our study.

### Conventional solvent extraction

The extracts of the corn silk powder were prepared by the maceration technique using the method proposed by Rusak et al. (17). Two different solvents namely aqueous methanol and water were utilized with three different time intervals (20, 30, and 40 min), temperatures (30, 40, and 50°C), and solvent-to-sample ratios (40:60, 60:40, and 80:20). The extracts obtained as a result of the technique used were further treated for concentration and was turned to powder form using the freeze dry technique and then stored at an optimum temperature (Table 1A).

**TABLE 1A:** Experimental design conditions for conventional solvent extraction.

Corn variety	Treatment	Solvent	Time (minutes)
Immature	T_1_	Water	30
	T_2_	Aqueous methanol	30
	T_3_	Water	60
	T_4_	Aqueous methanol	60
	T_5_	Water	90
	T_6_	Aqueous methanol	90
Mature	T_7_	Water	30
	T_8_	Aqueous methanol	30
	T_9_	Water	60
	T_10_	Aqueous methanol	60
	T_11_	Water	90
	T_12_	Aqueous methanol	90

### Supercritical fluid extraction (SFE)

The supercritical fluid extraction of polyphenols from the corn silk was carried out using three different factors including time, pressure, and temperature of 20, 30, and 40 min; 2,000, 3,000, and 4,000 Pa; and 30, 40, and 50°C, respectively (18), as shown in Table 1B.

**TABLE 1B:** Experimental design conditions for supercritical fluid extraction.

Independent variables	Code levels		
Temperature (°C)	30	60	90
Pressure (psi)	20	30	40
Time (minutes)	40:60	60:40	80:20

### Ultrasonic-assisted extraction (UAE)

The ultrasonic-assisted extraction of the corn silk was performed by the protocol given by Albu et al. (19) with some variations. The solvents utilized for the purpose of extraction were water and aqueous methanol at different time intervals (20, 30, and 40 min), temperatures (30, 40, and 50°C), solvent-to-sample ratio (40:60, 60:40, and 80:20), and amplitude (20, 30, and 40%) (Table 1C).

**TABLE 1C:** Experimental design conditions for ultrasonic-assisted extraction.

Independent variables	Code levels		
Amplitude (%)	30	60	90
Temperature (°C)	20	30	40
Solvent-to-sample ratio	40:60	60:40	80:20
Time (minutes)	5	10	15

### Phytochemical screening assays

Phytochemical screening of the extracts obtained from different techniques was carried out by performing various antioxidant assays including total phenolic content (TPC), total flavonoid content (TFC), 1,1-diphenyl-2-picrylhydrazyl (DPPH), 2,2′-azino-bis, 3-ethylbenzothiazoline-6-sulfonic acid (ABTS), and ferric-reducing antioxidant power (FRAP) estimation.

### Determination of total phenolics contents (TPC)

To determine the antioxidant potential of the extracts, the TPC was estimated through the assay against the standard reagents. For this purpose, an equal amount of the FC agent and the sample of corn silk was mixed with 500 μL of water (distilled) and allowed to rest for approximately 5 min after, 7% Na_2_CO_3_ (4.5 ml) was incorporated and allowed to rest for another 90 min. Finally, the assay was used to measure the absorbance through the spectrophotometer (IRMECO, U2020) at 760 nm. The TPC was then estimated as the gallic acid equivalent (mg gallic acid/g) using the method proposed by Sengul (20).

### Determination of total flavonoid (TF)

One of the important polyphenol classes includes flavonoids, which are the largest class and can provide therapeutic benefits. In the assay to determine the TFC, a mixture consisting of distilled water, NaNO_2_, and 10 percent AlCl_3_ in the ratio of 0.1, 4, 0.3, and 5%, respectively, was prepared. The solution was allowed to rest for 6 min and then 1.0M NaOH was added. The absorbance was then estimated at 430 nm following the protocol of Ghasemzadeh and Jaafar (21).

### Antioxidant potential

#### DPPH radical scavenging assay

The free radical scavenging ability was determined by the DPPH (1,1-diphenyl-2-picrylhydrazyl) assay. The estimation is carried out by taking the sample and the DPPH solution at 0.12 mM concentration added in a test tube in the ratio of 4:1, respectively. The solution was allowed to rest in a dark place for approximately 30 min. Later on, the absorbance of the solution was estimated at 520 nm using the UV/visible spectrophotometer against the standard control and the blank (22, 23).

#### ABTS (2,2′-azino-bis, 3-ethylbenzothiazoline-6-sulfonic acid) assay

The ABTS (2,2′-azino-bis, 3-ethylbenzothiazoline-6-sulfonic acid) assay of the corn silk extracts was carried out by the procedure proposed by Hossain et al. (24). To prepare the ABTS radicals, ABTS solution of 7 mM concentration was incorporated with 5 ml of potassium per sulfate solution of 2.45 mM concentration to make a total of 10 ml volume. This prepared mixture was transferred to an opaque bottle and allowed to rest for approximately 16 h in a dark and cool place to achieve stable oxidization. After the mixture had rested, it was diluted with ethanol and then adjusted to provide 0.7 absorbance at 734 nm. In addition, 10 μL of corn silk extract was added to 1 ml of the ABTS solution and then mixed thoroughly. After mixing, the solution was then subjected to a spectrophotometer to measure the absorbance at 734 nm after making it rest for 30 min. The antioxidant capacity of the extracts was determined using Trolox standard curve recorded in μmol Trolox/g sample extract.

#### Ferric-reducing antioxidant power (FRAP)

Another assay performed for the determination of the antioxidant capacity of the extract was the FRAP. In this assay, the metal ion chelating power is the parameter that helps in assessing the antioxidant potential of the compound under experiment. For this purpose, 0.5 ml of the experimental sample was added to 125 ml of the phosphate buffer with a concentration of 0.2 M and pH of 6.6, and potassium ferricyanide solution with a concentration of 1% was altogether placed in a water bath at a temperature of 50°C for 15 min. In the sample, 125 ml of trichloroacetic acid (10%) and distilled water were incorporated with 0.25 ml of ferric chloride (1%) and allowed to rest for 10 min. After the resting period, the absorbance was calculated at 700 nm (25).

#### HPLC quantification of bioactive compounds

The different preparations of corn silk extracts were then subjected to HPLC quantification to determine the comparative profusion of the bioactive compounds present. Ferulic acid which was found in abundance in corn silks has proven to be a potent antioxidant with health benefits. Purposely, the HPLC quantification was carried out for the approximation of ferulic acid content in corn silk extracts obtained through the extraction techniques mentioned above. Reverse phase HPLC (PerkinElmer, Series 200, USA) with C18 column was utilized. The mobile phase consisted of methanol/H_2_O, 65:35 (v/v), with a sample size of 1 ml, and at a per-minute flow rate of 1.0 ml. For the estimation procedure, ultraviolet detection was carried out at 282 nm. The comparison with the peak time and height of the ferulic acid standard was provided with the calculations (26).

#### Statistical analysis

The data for each parameter was statistically analyzed to determine the level of significance using a software package (MATLAB). Analysis of variance technique (ANOVA) was applied for the estimation of significance (*p* ≤ 0.05) between the various factors. Purposely, a one-factor or two-factor factorial design was applied as per the nature of the variables. Moreover, the comparison of the extraction techniques and maturity vs. immaturity two-factor factorial design was applied. Duncan’s multiple ranges (DMR) test was utilized to estimate the level of significance between the groups.

## Results and discussion

### Nutritional composition of corn silk

#### Proximate and mineral analysis

The mean values for moisture, protein, fat, fiber, total ash, and NFE (nitrogen-free extract) of immature and mature corn silk were found to be 5.25 ± 1.12 and 4.40 ± 2.19, 12.83 ± 0.89 and 8.75 ± 0.57, 1.23 ± 0.02 and 0.54 ± 0.01, 49.51 ± 3.91 and 53.25 ± 2.89, 5.38 ± 0.19 and 5.49 ± 0.22, and 25.80 ± 1.19 and 27.57 ± 1.56%, respectively. Likewise, the values observed for minerals in immature and mature corn silk were sodium (Na) 193.47 ± 5.58 and 289.31 ± 4.16, potassium (K) 2,629.97 ± 4.82 and 3,560.24 ± 5.96, calcium (Ca) 1,083.09 ± 6.17 and 709.94 ± 4.38, magnesium (Mg) 1,213.19 ± 5.45 and 359.90 ± 2.29, zinc (Zn) 42.46 ± 2.28 and 36.78 ± 1.99, manganese (Mn) 34.14 ± 1.79 and 33.57 ± 2.53, and iron (Fe) 2.13 ± 0.08 and 4.39 ± 1.11 μg/g, respectively (Table 2).

**TABLE 2:** Compositional analysis of corn silk.

Nutritional compound	Concentration	
	Immature corn silk	Mature corn silk
Moisture (%)	5.25 ± 1.12a	4.40 ± 2.19b
Protein (%)	12.83 ± 0.89a	8.75 ± 0.57b
Fat (%)	1.23 ± 0.02a	0.54 ± 0.01b
Fiber (%)	49.51 ± 3.91b	53.25 ± 2.89a
Ash (%)	5.38 ± 0.19b	5.49 ± 0.22a
NFE	25.80 ± 1.19b	27.57 ± 1.56a
Na (μg/g)	193.47 ± 5.58b	289.31 ± 4.16a
Ca (μg/g)	1,083.09 ± 6.17a	709.94 ± 4.38b
Mg (μg/g)	1,213.19 ± 5.45a	359.90 ± 2.29b
K (μg/g)	2,629.97 ± 4.82b	3,560.24 ± 5.96a
Cu (μg/g)	6.42 ± 1.20a	4.59 ± 0.92b
Fe (μg/g)	2.13 ± 0.08b	4.39 ± 1.11a
Zn (μg/g)	42.46 ± 2.28a	36.78 ± 1.99b
Mn (μg/g)	34.14 ± 1.79a	33.57 ± 2.53b

Values are expressed as mean ± standard deviation (n = 3). Values in the same column within each parameter with different letters were significantly different from each other (p ≤ 0.05).

#### *In vitro* characterization

The grand mean obtained for the parameters of the *in vitro* characterization including TPC, TFC, radical scavenging assay (DPPH), ferric-reducing antioxidant potential (FRAP), and ABTS assay for the mature and immature corn silk extracts showed that both the extraction techniques [(*p* ≤ 0.05)] and stage of maturity of corn silk [(*p* ≤ 0.05)] showed significant differences in the antioxidant profile, whereas their interaction showed non-significant differences [(*p* 0.0250)]. Moreover, the effect of the time, temperature, type of solvent used, pressure, and amplitude was found to be quite significant on the TPC, TFC, DPPH, FRAP, and ABTS of the obtained extracts. The peak recovery was presented by the supercritical fluid extraction followed by the ultrasound, and the least was found to be by the conventional extraction. The peak values were obtained in the supercritical fluid extraction with the parameters including a pressure of 4,000 Pa, temperature of 50°C, and time interval of 40 min, whereas in the ultrasonic extraction, the highest recovery point was with the following parameters: a temperature of 50°C, a time interval of 40 min, ethanol of 80%, and an amplitude of 40%. Similarly, in the conventional extraction technique, the parameters included 50°C temperature, 40 min time interval, and 80% ethanolic solution, which showed better recovery as compared to other solvents utilized (Table 3).

**TABLE 3:** Comparison of extraction techniques on the collective antioxidant profile of polyphenols from immature and mature corn silk polyphenols.

Extraction module	Immature corn silk	Mature corn silk	Mean
Conventional solvent extraction	48.54 ± 1.12	41.38 ± 1.10	44.96 ± 1.19C
Ultrasound-assisted extraction	55.74 ± 2.22	51.16 ± 1.45	53.45 ± 1.81B
Supercritical fluid extraction	76.88 ± 2.24	62.28 ± 1.01	69.58 ± 1.90A
	60.39 ± 3.31A	51.61 ± 2.20B	

Values are expressed as mean ± standard deviation (n = 3). Values in the same column within each parameter with different letters were significantly different from each other (p ≤ 0.05). Two-way repeated measure ANOVA showed a significant effect of the extraction techniques [F(112.17) = p < 0.001], a significant effect of the stage of picking [(40.31) p = 0.0000], and a non-significant interaction between factors (F5.10) = p = 0.0250).

#### Antioxidant potential of mature and immature corn silk extracts

The below tables represent the effect of treatments on TPC, TFC, DPPH, FRAP, and ABTS assays of the mature and immature corn silk extracts, respectively. The treatment with the highest TPC value was the supercritical fluid extract, while lower values were observed in the ultrasonicated extraction technique (Table 4). Further decreased values were found to be for the conventionally extracted extracts. A similar trend was observed for the TFC, which showed the peak values for the supercritical fluid extraction to be the same as the former. Also seen in the DPPH (Table 5) and FRAP (Table 6) was the same trend as the TPC, showing maximum extraction in the supercritical fluid technique (T_9_S_2_), followed by the ultrasound (T_6_S_1_ and T_6_S_2_) and conventional (T_3_S_1_ and T_3_S_2_) techniques. The highest mean values for the TPC of the mature and immature corn silk extracts displayed by the supercritical fluid extraction presented the values to be 143.8917 ± 2.32 (immature) and 112.2672 ± 2.07 (mature) mg/GAE 100 g for the extract T_9_S_2_. Similar results were obtained for TFCs (Table 7) for the extract T_9_S_2_ to be 97.0109 ± 1.99 (immature) and 76.45491 ± 2.22 (mature) mg CE/100 g. The values of DPPH, FRAP, and ABTS (Table 8) showed the same trend followed by the TPC and TFC as mentioned earlier. The values of DPPH were found to be 117.0109 ± 4.52% (immature) and 96.45491 ± 2.57% (mature), that of FRAP were noted to be 80.64263 ± 2.11 (immature) and 66.41158 ± 1.90 (mature) μM (Fe + 2/g), while that of ABTS were presented as 1.206477 ± 0.228 (immature) and 1.200152 ± 0.902 (mature) μM TE/g.

**TABLE 4:** Comparison of total phenolic contents (mg/100 g gallic acid equivalent) of mature and immature corn silk extracted through different extraction technologies.

Treatments	Immature corn silk	Mature corn silk	Mean
**Conventional**			
T1S1	75.01 ± 4.09	64.12 ± 2.01	69.50 ± 1.90F
T1S2	95.29 ± 3.28	81.31 ± 2.22	88.30 ± 3.02C
T2S1	78.75 ± 1.08	67.20 ± 2.77	72.98 ± 2.65E
T2S2	104.82 ± 1.80	89.44 ± 1.95	97.13 ± 2.41B
T3S1	86.63 ± 2.70	73.92 ± 2.38	80.27 ± 1.72D
T3S2	115.30 ± 2.59	98.39 ± 2.62	106.84 ± 2.77A
Mean	92.63 ± 6.25A	79.06 ± 3.21B	
**Ultrasound**			
T4s1	85.18 ± 1.05	69.23 ± 0.49	77.91 ± 0.67D
T4s2	104.13 ± 3.75	84.53 ± 1.59	94.33 ± 1.29B
T5S1	97.32 ± 0.96	73.83 ± 0.91	82.39 ± 0.72CD
T5S2	111.42 ± 4.67	90.45 ± 0.68	100.93 ± 2.41AB
T6S1	97.32 ± 2.77	78.10 ± 2.38	88.16 ± 1.95C
T6S2	119.22 ± 2.66	96.78 ± 2.34	107.10 ± 2.75A
Mean	102.43 ± 2.25A	82.15 ± 4.21B	
**Supercritical fluid extraction**			
T7S2	118.99 ± 5.75	92.78 ± 1.55	105.85 ± 1.73B
T8S2	130.81 ± 3.30	102.06 ± 2.47	116.44 ± 3.89AB
T9S2	143.89 ± 2.32	112.27 ± 2.07	128.08 ± 3.74A
Mean	131.23 ± 6.45A	102.37 ± 3.25B	

Values are expressed as mean ± standard deviation (n = 3). Values in the same column within each parameter with different letters were significantly different from each other (p ≤ 0.05).

**TABLE 5:** Comparison of DPPH activity (%) of mature and immature corn silk extracted through different extraction technologies.

Treatments	Immature corn silk	Mature corn silk	Mean
**Conventional**			
T1S1	55.16 ± 1.75	49.97 ± 1.82	52.28 ± 2.20E
T1S2	69.88 ± 0.38	62.25 ± 1.44	66.07 ± 2.18C
T2S1	57.75 ± 0.27	51.45 ± 0.84	54.60 ± 1.55ED
T2S2	76.87 ± 0.49	68.48 ± 0.76	72.67 ± 0.96AB
T3S1	63.52 ± 1.71	56.60 ± 0.43	60.06 ± 1.02D
T3S2	84.55 ± 1.15	75.33 ± 1.14	79.94 ± 0.96A
Mean	67.95 ± 1.01A	60.68 ± 2.20B	
**Ultrasound**			
T4s1	65.25 ± 1.15	55.02 ± 2.48	60.61 ± 1.50C
T4s2	69.84 ± 1.69	67.38 ± 1.17	68.61 ± 1.41AB
T5S1	61.90 ± 0.80	58.85 ± 1.08	59.93 ± 1.90C
T5S2	74.73 ± 0.78	72.09 ± 0.79	73.41 ± 0.51A
T6S1	65.27 ± 0.58	62.97 ± 0.79	64.12 ± 0.72B
T6S2	79.96 ± 0.41	77.14 ± 0.64	78.55 ± 0.28A
Mean	69.49 ± 1.23A	55.57 ± 2.90B	
**Supercritical fluid extraction**			
T7S2	96.70 ± 2.04	79.71 ± 0.62	88.21 ± 0.52C
T8S2	106.37 ± 1.52	87.69 ± 1.94	97.02 ± 1.45B
T9S2	117.01 ± 4.52	96.45 ± 2.57	106.73 ± 5.10A
Mean	106.69 ± 5.15A	87.95 ± 3.18B	

Values are expressed as mean ± standard deviation (n = 3). Values in the same column within each parameter with different letters were significantly different from each other (p ≤ 0.05).

**TABLE 6:** Comparison of FRAP (μM Fe + 2/g) of mature and immature corn silk extracted through different extraction technologies.

Treatments	Immature corn silk	Mature corn silk	Mean
**Conventional**			
T1S1	35.17 ± 0.24	28.65 ± 0.62	31.51 ± 0.52E
T1S2	44.47 ± 1.69	35.57 ± 0.84	40.02 ± 1.55C
T2S1	36.75 ± 0.79	29.48 ± 0.50	33.08 ± 0.78D
T2S2	48.91 ± 2.18	39.13 ± 0.51	44.02 ± 0.38B
T3S1	40.43 ± 1.55	32.34 ± 0.31	36.38 ± 1.08CD
T3S2	53.81 ± 1.41	43.04 ± 0.81	48.43 ± 1.14A
Mean	43.25 ± 2.29A	34.70 ± 1.10B	
**Ultrasound**			
T4s1	45.15 ± 0.84	38.44 ± 0.27	41.59 ± 0.89D
T4s2	69.84 ± 0.43	76.71 ± 1.15	73.27 ± 0.66B
T5S1	61.87 ± 1.47	67.46 ± 1.44	64.11 ± 1.55C
T5S2	74.73 ± 1.90	82.08 ± 2.01	78.40 ± 0.97AB
T6S1	65.28 ± 0.99	71.69 ± 0.79	68.48 ± 0.88BC
T6S2	79.96 ± 1.22	87.82 ± 1.98	83.89 ± 2.03A
Mean	66.13 ± 1.10A	70.70 ± 3.10B	
**Supercritical fluid extraction**			
T7S2	66.65 ± 0.99	54.89 ± 2.30	60.77 ± 1.55C
T8S2	73.31 ± 1.67	60.37 ± 1.92	66.84 ± 0.97B
T9S2	80.64 ± 2.11	66.41 ± 1.90	73.53 ± 2.33A
Mean	73.53 ± 1.04A	60.55 ± 2.20B	

Values are expressed as mean ± standard deviation (n = 3). Values in the same column within each parameter with different letters were significantly different from each other (p ≤ 0.05).

**TABLE 7:** Comparison of total flavonoid contents (mg CE/100 g) of mature and immature corn silk extracted through different extraction technologies.

Treatments	Immature corn silk	Mature corn silk	Mean
**Conventional**			
T1S1	25.91 ± 0.24	19.79 ± 0.55	22.55 ± 0.19D
T1S2	39.88 ± 0.99	32.25 ± 0.89	36.07 ± 0.28B
T2S1	27.75 ± 0.24	21.45 ± 0.56	24.61 ± 0.33CD
T2S2	46.87 ± 1.09	38.48 ± 0.45	42.67 ± 1.90AB
T3S1	33.53 ± 0.77	26.60 ± 0.71	30.06 ± 0.22C
T3S2	54.55 ± 1.02	45.33 ± 0.67	49.94 ± 0.11A
Mean	30.08 ± 1.12A	30.65 ± 1.20B	
**Ultrasound**			
T4s1	35.61 ± 0.09	25.29 ± 0.88	30.17 ± 0.19D
T4s2	39.84 ± 0.18	37.38 ± 1.11	38.61 ± 0.89B
T5S1	31.90 ± 0.76	28.85 ± 0.58	29.93 ± 0.67
T5S2	44.73 ± 1.09	42.09 ± 1.89	43.41 ± 1.20A
T6S1	35.27 ± 1.22	32.97 ± 1.90	34.12 ± 0.28C
T6S2	49.96 ± 1.88	47.14 ± 1.35	48.55 ± 1.06A
Mean	39.55 ± 1.06A	35.62 ± 2.20B	
**Supercritical fluid extraction**			
T7S2	66.70 ± 2.90	49.71 ± 1.38	58.20 ± 1.99C
T8S2	76.37 ± 2.33	67.69 ± 2.10	72.03 ± 2.91B
T9S2	97.01 ± 1.99	76.45 ± 2.22	86.73 ± 2.75A
Mean	80.02 ± 1.02A	64.61 ± 2.20B	

Values are expressed as mean ± standard deviation (n = 3). Values in the same column within each parameter with different letters were significantly different from each other (p ≤ 0.05).

**TABLE 8:** Comparison of ABTS (μM TE/g) of mature and immature corn silk extracted through different extraction technologies.

Treatments	Immature corn silk	Mature corn silk	Mean
**Conventional**			
T1S1	0.746 ± 0.006	1.483 ± 0.002	1.115 ± 0.005F
T1S2	0.948 ± 0.007	1.884 ± 0.004	1.416 ± 0.019E
T2S1	0.783 ± 0.008	1.557 ± 0.029	1.170 ± 0.001D
T2S2	1.043 ± 0.002	2.073 ± 0.005	1.558 ± 0.010C
T3S1	0.8620.003	1.713 ± 0.040	1.287 ± 0.007B
T3S2	1.147 ± 0.012	2.280 ± 0.009	1.713 ± 0.019A
Mean	0.921 ± 0.01A	1.831 ± 0.03B	
**Ultrasound**			
T4s1	0.756 ± 0.018	1.423 ± 0.014	1.0895 ± 0.001E
T4s2	1.091 ± 0.023	2.218 ± 0.091	1.651 ± 0.005B
T5S1	0.897 ± 0.109	1.595 ± 0.004	1.246 ± 0.128D
T5S2	1.221 ± 0.120	1.193 ± 0.008	1.207 ± 0.002D
T6S1	0.901 ± 0.017	2.091 ± 0.156	1.496 ± 0.172C
T6S2	1.998 ± 0.001	2.266 ± 0.029	2.132 ± 0.034A
Mean	1.144 ± 0.10A	1.920 ± 0.05B	
**Supercritical fluid extraction**			
T7S2	0.997 ± 0.090	0.992 ± 0.078	0.994 ± 0.109C
T8S2	1.097 ± 0.219	1.091 ± 0.277	1.094 ± 0.781B
T9S2	1.206 ± 0.228	1.200 ± 0.902	1.203 ± 0.182A
Mean	1.10 ± 0.04A	1.09 ± 0.01B	

Values are expressed as mean ± standard deviation (n = 3). Values in the same column within each parameter with different letters were significantly different from each other (p ≤ 0.05).

#### HPLC quantification of ferulic acid in corn silk

**FIGURE 1:**
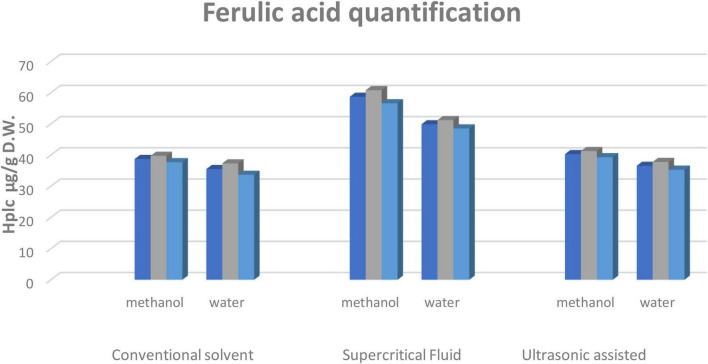
HPLC quantification of ferulic acid from corn silks. Values for ferulic acid characterized through reverse phase HPLC in μg/g. Two solvents and three extraction techniques were applied. Values are mean ± SEM (*n* = 03) and the level of significance was determined at (*p* ≤ 0.05).

The statistical analysis of corn silk bioactive components displayed considerable differences in the ferulic acid content of the extracts obtained through different techniques. The varying solvents i.e., methanol and water, and their respective ratios showed ferulic acid as having the highest recovery in aqueous methanolic extract among all the techniques. Among the extraction techniques, supercritical fluid extraction showed the highest quantity of ferulic acid, compared to the conventional and UAE techniques (Figure 1).

## Discussion

This research might be the first to highlight the impact of different extraction techniques (supercritical fluid, ultrasonication, and conventional) and their parameters of extraction and isolation of corn silk polyphenols. Moreover, their maturity and immaturity were also discussed according to the different extraction techniques in the light of the study. The results of the current study found an ethanolic solution for extraction to be much more effective, compared to the other solvents. Similar results, as in the study conducted by Humadi and Istudor (27), were obtained for the extracts obtained from a methanolic solution, which displayed lower TPCs in both the mature and the immature corn silk extracts when compared to the aqueous extracts, noting the values to be 40.38 + 1.10 μg/g of TAE lower than 42.71 + 0.87 μg/g of TAE. The extraction procedure involved in this ongoing study utilized different solvents and techniques as in the study conducted by Das et al. (28). The use of different solvents led to greater extractions of the required polyphenols, which elevated the antioxidant potential of the corn silk extracts (29, 30). Moreover, the affinity of the phenols for similar polarity solvents plays an important role in extraction processes (31). The possibility of having lower antioxidants might be due to the limitations of utilizing the whole corn silks due to hygienic issues; however, the yellow color imparting corn silks providing the flavones and the flavonols can be of great importance. The polyphenols which present as antioxidants also play a vital role in chelating metals and scavenging free radicals (32–34). On the other hand, there can still be several factors like fertilization, soil conditions, size, maturity, season, etc., which may affect the antioxidant potential of corn silk. According to earlier studies (35, 36), the ethanolic method is greatly used for extraction purposes. The extraction of the polyphenols and the flavonoids depended on the different extraction conditions including extraction time and extraction temperature, as well as the pressure and amplitude in some of the extraction techniques (34, 37, 38). Insufficient time for extraction may yield lower concentrations of flavonoids; thus, it is best to have an optimum temperature and time for carrying out the proper extraction procedure. In a study carried out earlier on the optimum condition for the extraction of corn silks, it was found that 5.32 mg of the total flavonoids was extracted from 1 g of corn silk (39). The different polarities of different solvents allowed for the diffusion of various constituents of the material in the plant and thus helped with the extraction yield as well. In a previous investigation, the percentage of the radical scavenging activity (DPPH) was observed to rise with larger concentrations of the extract in all samples. The methanolic extract had the greatest DPPH activity in the research, followed by the ethanolic and aqueous extracts. The values noted were 140.89 μg/ml, 143.55 μg/ml, and 195.21 μg/ml, respectively.

## Conclusion and recommendations

With the advancement of technology, the extraction from several plant varieties has become quite easier than in the earlier days. The wide use of microwave-assisted, ultrasound, and supercritical fluid extraction is now taking the place of the conventional techniques used in older days. Utilizing plant sources for their beneficial antioxidants has been carried out around the world by several researchers and scientists. Our study comprising of the extraction of polyphenols from the corn silk has provided new insight into the beneficial aspects of the rarely used part, the corn silk of the corn scientifically known as the *Zea mays*. The study of the antioxidant potential of these corn hairs or the corn silk has displayed a potent scavenging free radical effect, chelating of the catalytic metal ions, and also exerting a therapeutic effect against the oxidative stress/damage to the cellular macromolecules. The chemical examination of the different varieties of extracts showed the presence of polyphenols and flavonoids, imposing the antioxidant characteristic of the plant material thereof. The greater scavenging power of the corn silk may be possible because of the hydroxyl group that exists in the phenolic compounds. Thus, these seldomly utilized parts of the corn (*Zea mays* L.) named corn silk can be of great importance due to their antioxidant potential. From a future perspective, extensive characterization of the by-products through GC-MR spectroscopy can be carried out for elucidating the potential polyphenolics for further utilization.

## Data availability statement

The raw data supporting the conclusions of this article will be made available by the authors, without undue reservation.

## Author contributions

NF: conceptualization and writing—original draft. AI: supervision, writing—original draft and review, and editing. MUA: validation and methodology. MSA: formal analysis, investigation, and resources. All authors have read and agreed to the published version of the manuscript.
